# Metagenomic profiling of historic Colorado Front Range flood impact on distribution of riverine antibiotic resistance genes

**DOI:** 10.1038/srep38432

**Published:** 2016-12-05

**Authors:** Emily Garner, Joshua S. Wallace, Gustavo Arango Argoty, Caitlin Wilkinson, Nicole Fahrenfeld, Lenwood S. Heath, Liqing Zhang, Mazdak Arabi, Diana S. Aga, Amy Pruden

**Affiliations:** 1 Department of Civil and Environmental Engineering, Virginia Tech, Blacksburg, VA 24061, USA; 2 Department of Chemistry, University at Buffalo, The State University of New York, Buffalo, NY 14260, USA; 3 Department of Computer Science, Virginia Tech, Blacksburg, VA 24061, USA; 4 Department of Civil and Environmental Engineering, Rutgers University, Piscataway, NJ 08854, USA; 5 Department of Civil and Environmental Engineering, Colorado State University, Fort Collins, CO, 80523, USA

## Abstract

Record-breaking floods in September 2013 caused massive damage to homes and infrastructure across the Colorado Front Range and heavily impacted the Cache La Poudre River watershed. Given the unique nature of this watershed as a test-bed for tracking environmental pathways of antibiotic resistance gene (ARG) dissemination, we sought to determine the impact of extreme flooding on ARG reservoirs in river water and sediment. We utilized high-throughput DNA sequencing to obtain metagenomic profiles of ARGs before and after flooding, and investigated 23 antibiotics and 14 metals as putative selective agents during post-flood recovery. With 277 ARG subtypes identified across samples, total bulk water ARGs decreased following the flood but recovered to near pre-flood abundances by ten months post-flood at both a pristine site and at a site historically heavily influenced by wastewater treatment plants and animal feeding operations. Network analysis of *de novo* assembled sequencing reads into 52,556 scaffolds identified ARGs likely located on mobile genetic elements, with up to 11 ARGs per plasmid-associated scaffold. Bulk water bacterial phylogeny correlated with ARG profiles while sediment phylogeny varied along the river’s anthropogenic gradient. This rare flood afforded the opportunity to gain deeper insight into factors influencing the spread of ARGs in watersheds.

In September 2013, historic flooding impacted the Colorado Front Range, with some locations experiencing a rare 1-in-1,000 year rainfall event^1^. A record-setting flood peak of 3.3 m was recorded for the Cache La Poudre (Poudre) River in Fort Collins, resulting in a major transformation of the watershed landscape and massive transport of sediment throughout the basin^2^. Since 2002, the Poudre River has served as a field observatory for characterizing the impact of urban and agricultural activities on antibiotics and antibiotic resistance genes (ARGs)^3^,^4^,^5^,^6^,^7^. The distinct gradient of anthropogenic influence as the Poudre River flows from its pristine origin in the Rocky Mountains to areas heavily impacted by animal feedings operations (AFOs) and wastewater treatment plants (WWTPs) has previously served to demonstrate that human activities significantly alter ARG occurrence in river bed sediment and bulk water^7^. In particular, the upstream capacity of AFOs and WWTPs, weighted to account for the inverse distance of these facilities from riverine sampling sites, strongly correlated (R^2^ = 0.92) with *sul*1, marking it as a key indicator of human influence.

Antibiotic resistance presents a critical challenge to public health. While antibiotic resistance is a natural capability among many bacteria, with diverse ARGs profiled even in remote and ancient soils and caves^8^,^9^,^10^,^11^,^12^, widespread use of antibiotics both in livestock and humans is linked with increased frequency of resistant infections reported in clinical settings and increased ARG abundance in aquatic and terrestrial environments^9^,^13^,^14^,^15^,^16^,^17^. Through comparison of DNA sequence similarity, instances have been identified in which pathogens likely obtained ARGs from environmental reservoirs^18^,^19^. Though gene transfer events of ARGs from environmental bacteria to human pathogens are thought to be rare^20^, the consequences can be devastating. For example, the recent emergence and spread of *bla*_NDM-1_, which is frequently found on a genetic element carrying several genes conferring resistance to multiple antibiotics, is thought to have originated via horizontal transfer from a plant pathogen to a human pathogen^21^.

Surface water is now well-documented as a receiving environment for anthropogenic sources of ARGs and also represents a critical linkage back to humans both as a recreational and drinking water resource^7^,^22^,^23^,^24^,^25^. The factors governing the dissemination of ARGs in watersheds are complex and not well understood. In particular, transport of resistant bacteria and ARGs from human sources, such as WWTPs and AFOs, selection of allochthonous and authochthonous resistant bacteria by antibiotics and other agents, and horizontal gene transfer have been cited as key mechanisms governing the proliferation of ARGs in watersheds^22^,^26^,^27^. Contamination with antibiotics is of particular interest as they could exert direct or co-selective pressures on ARGs of different classes and also stimulate horizontal gene transfer^28^,^29^,^30^,^31^,^32^. Likewise, various metals can also stimulate the latter two processes^14^,^33^,^34^,^35^,^36^, though few studies have elucidated the relationship between occurrence of ARGs and antibiotics or metals in surface water. Understanding the relative roles of antibiotics and metals in proliferating antibiotic resistance in the environment is important for developing effective management guidelines for antimicrobial use and management of urban and agricultural waste streams.

In the wake of unprecedented rainfall in the Poudre River basin, we sought to characterize the impact of flooding and subsequent recovery on the occurrence of ARGs and examine the influence of antibiotics and metals. We annotated shotgun metagenomic reads against existing ARG and heavy metal resistance gene (MRG) databases to profile the resistome of pristine and heavily impacted sites before and after the flood. Correlations of select ARGs, quantified by quantitative polymerase chain reaction (qPCR), with antibiotics and heavy metals were examined. Amplicon sequencing of 16S rRNA genes enabled comparison of the resistome with the microbial phylogenetic composition as an indicator of the relative importance of vertical gene transfer and physical transport of bacteria. To explore the role of horizontal gene transfer in shaping the resistome, metagenomic reads were annotated against a mobile genetic element database. Network analysis of *de novo* assembled metagenomic scaffolds revealed ARGs exhibiting physical genetic linkages to mobile genetic elements, MRGs, and other ARGs. Comprehensive profiling of ARGs and factors hypothesized to contribute to their selection, proliferation, and spread in the environment before and after an extreme flooding event provided unique insight into the mechanisms governing the dissemination of ARGs in the water environment. This knowledge will be particularly important in upcoming decades when the frequency and severity of storms is expected to increase as a consequence of climate change^37^.

## Materials and Methods

### Sample Collection and Preservation

**Figure 1:**
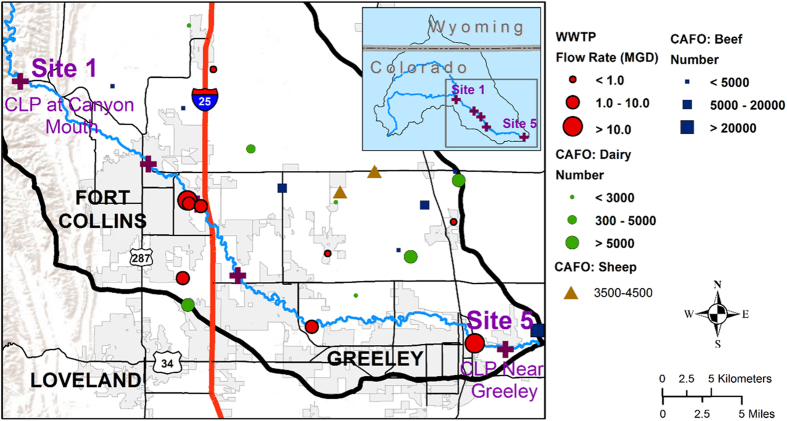
Poudre River sampling sites. Contributing wastewater treatment plants (WWTPs) and animal feeding operations (AFO) are indicated with their respective capacities in million gallons per day (MGD) and animal counts. The figure was created by co-author Mazdak Arabi using the ArcGIS software by ESRI, Release 10.1 (ESRI, Redlands, CA) (http://www.esri.com/software/arcgis).

Bulk water, including the suspended sediment therein, and bed sediment samples were collected from five previously described river sites, representing a gradient of anthropogenic influence, as well as from a WWTP that discharges into the river^5^. Briefly, site 1 is a pristine location near the river’s origin in the Rocky Mountains, site 2 is upstream of Fort Collins and receives light agricultural runoff, site 3 is within Fort Collins and receives agricultural and urban stormwater runoff, site 4 is downstream of two WWTPs, (combined average effluent: 42,000 m^3^/d), and site 5 is downstream of Fort Collins and Greeley and is heavily impacted by adjacent agricultural and urban land use and a 28,000 m^3^/d WWTP (Fig. 1). Bulk water was collected from the center of the flow channel in sterile 1-L polypropylene containers for molecular analysis and in 1-L amber glass bottles pretreated as described by Tso *et al*.^38^ for antimicrobial analysis. Duplicate bulk water samples for metal analysis were collected in 50-mL metal-free polypropylene centrifuge tubes. Triplicate sediment samples (~30 g) were collected from the top 5 cm of bed sediment using a sterile spade for molecular analysis. Water quality information was collected using a Hydrolab MS5 multiparameter sonde (OTT Hydromet, Loveland, CO). Samples were transported to the lab on ice and preserved within 24 hours of collection.

Bulk water samples for molecular analysis were concentrated onto 0.22 μm mixed cellulose esters membrane filters (Millipore, Billerica, MA). Filters were folded into quarters and cut into 1 cm^2^ pieces using a sterile blade and transferred to extraction tubes. Sediment was homogenized and 0.5 g was transferred to extraction tubes. DNA was extracted using a FastDNA SPIN Kit for Soil (MP Biomedicals, Solon, OH). A filter blank and DNA extraction blank were also extracted.

Samples for antimicrobial analysis were preserved according to Tso *et al*.^38^. The method was modified to use a surrogate solution containing d_4_-sulfamethoxazole, ^13^C_6_-sulfamethazine, ^13^C-erythromycin, and demeclocycline (500 ng/mL). Samples for metal analysis were acidified to 2% (v/v) with fuming nitric acid and filtered through a 0.45 μm polypropylene syringe filter. A 10-mL aliquot was transferred to two 15-mL metal-free polypropylene centrifuge tubes. One aliquot was spiked with 50 uL of 1000 ng/mL spiking solution made from certified metal standards (BDH Aristar® PLUS 82026-108, 82026-100, respectively, VWR, Inc. Radnor, PA, USA). An equal volume of 2% nitric acid in water (v/v) was added to the remaining aliquot for quantification by single-point standard addition.

### Quantification of ARGs

Gene markers were quantified in triplicate reactions from DNA extracts using qPCR, with previously published protocols for 16S rRNA genes^39^ and five ARGs: *sul*1^6^, *sul*2^6^, *tet*(O)^40^, *tet*(W)^40^, and *erm*F^41^. Extracts were diluted between 1:10–1:50 to minimize inhibition. Triplicate standard curves of ten-fold serial diluted standards of each target gene ranging from 10^8^ to 10^2^ gene copies/μl for 16S rRNA and 10^7^ to 10^1^ gene copies/μl for ARGs were included for each run, along with a triplicate negative control. The limit of quantification was established as the lowest standard that amplified in triplicate in each run, ranging from 0.7 to 3.3 log gene copies/ml for bulk water and 3.6 to 6.0 log gene copies/g for sediment, depending on the gene assay and the measured volume or mass of sample.

### Quantification of Antibiotics and Metals

Antibiotics were quantified by liquid chromatography-tandem mass spectrometry (LC-MS/MS) as previously described for sulfonamides and tetracyclines^38^. A separate LC-MS/MS method was adapted from Wallace and Aga^42^ for macrolide antibiotics to enhance sensitivity. All analytes were normalized to the internal standard d_10_-carbamazepine (sulfonamides and macrolides) or minocycline (tetracyclines). Metals were quantified by inductively coupled plasma mass spectrometry (ICP-MS) on an X-Series 2 instrument (Thermo Scientific, Waltham, MA) using collision cell technology to reduce polyatomic interferences. Analytes were quantified using single-point standard addition and confirmed by external calibration curve (0.5 to 1000 ng/mL). The concentrations of Cr, Mn, Cu, As, Sr, Ag, and Cd were quantified using the most abundant isotope, normalized to the internal standard ^115^In. Barium, Ce, Gd, Pt, Pb, Th, and U were quantified analogously via a separate injection to maximize scan time for accurate quantification, normalized to internal standard, ^159^Tb.

### 16S rRNA Gene Amplicon Sequencing and Metagenomic Analysis

To explore the composition of the bacterial communities in bulk water and bed sediment, gene amplicon sequencing was conducted using barcoded primers (515 f/806r) designed to target the V4 region of the 16S rRNA gene^43^,^44^. Triplicate PCR products were composited, and 240 ng of each composite was combined and purified using a QIAquick PCR Purification Kit (Qiagen, Valencia, CA). Sequencing was conducted at the Virginia Bioinformatics Institute (VBI) Genomics Research Laboratory (Blacksburg, VA) on an Illumina MiSeq using a 250-cycle paired-end protocol. Processing of reads was conducted using the QIIME pipeline^45^ and annotation against the Greengenes database^46^ (May 2013 release). After quality filtering, between 37,150–444,433 reads were obtained per sample and all samples were rarefied to 37,150 randomly selected reads.

Shotgun metagenomics were conducted on water samples collected at sites 1 and 5, 12 months pre-flood and 3 and 10 months post-flood. Samples were prepared using the Nextera XT library prep (Illumina, San Diego, CA) and sequenced on an Illumina HiSeq 2500 using a 100-cycle paired-end protocol at VBI. Paired ends reads were merged using FLASH^47^. Quality filtering was conducted using Trimmomatic^48^ according to default parameters. Relative abundances were calculated by normalizing gene counts to abundance of 16S rRNA genes, as well as target gene and 16S rRNA gene length as proposed by Li *et al*.^49^. Absolute abundances were calculated by multiplying relative abundance of ARGs by total abundance of 16S rRNA genes, quantified by qPCR, as noted above. 16S rRNA genes were annotated using BLASTN^50^ against the Silva ribosomal RNA database^51^ (version 123). ARGs were annotated against the subset download of the Comprehensive Antibiotic Resistance Database^52^, which excludes genes that confer resistance via specific mutations (accessed August 2015). MRGs were annotated from the BacMet antibacterial biocide and metal resistance genes database^53^ (version 1.1), and proteins specific to the mobile genetic elements plasmids and prophages were annotated from the ACLAME database^54^ (version 0.4). Annotations made against the ACLAME database were manually screened to ensure known ARGs were not included. Functional gene annotation was performed using the DIAMOND protein aligner^55^ with a best hit approach using an amino acid identity cutoff of 90%, minimum alignment length of 25 amino acids, and 1e-5 e-value cutoff. Sequences were assembled prior to network analysis using the IDBA-UD *de novo* assembler^56^ and annotated using DIAMOND with a 1e-5 e-value cutoff. Unassembled sequences were uploaded to MG-RAST^57^ and are publicly available under accession numbers 462880.3–4628878.3 (Table S1).

### Statistical Analyses

Spearman’s Rank Correlation Coefficients were calculated in JMP to assess correlations between ARGs and metals, antibiotics, and water quality parameters using a significance cutoff of α = 0.05. UniFrac distances generated in QIIME were imported into PRIMER-E (version 6.1.13) for one-way analysis of similarities (ANOSIM). Metagenomic ARG relative abundances were imported into PRIMER-E and Bray-Curtis distances were used to generate multidimensional scaling plots. This distance matrix was compared with weighted UniFrac similarities for 16S rRNA gene amplicon sequencing using 2STAGE in PRIMER-E. Network analysis visualization was conducted using Gephi (version 0.8.2).

## Results and Discussion

### Metagenomic analysis reveals shift in ARG profile following extreme flooding event

**Figure 2:**
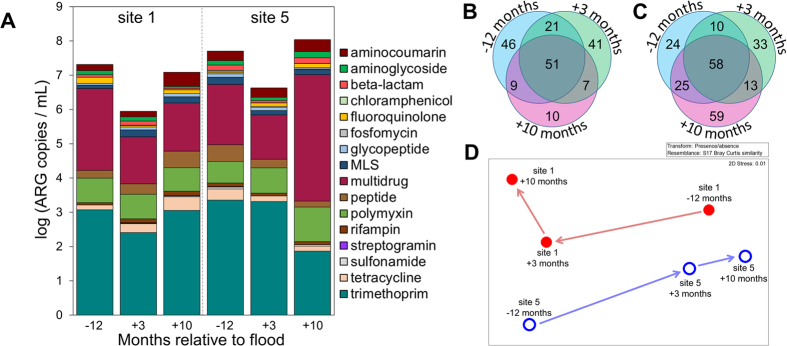
(**A**) Absolute abundance of ARGs by class per mL bulk water identified from metagenomic sequencing reads annotated against the Comprehensive Antibiotic Resistance Database. ARGs conferring resistance to two or more of the classes macrolide, lincosamide, and streptogramin are denoted as MLS. Venn diagrams represent number of ARGs unique and shared amongst sample dates indicated in number of months relative to flood at (**B**) site 1 and (**C**) site 5. (**D**) Nonmetric multidimensional scaling (NMDS) plot generated from Bray-Curtis similarity matrix of metagenomic ARG composition by date at site 1 (historically pristine) and site 5 (historically impacted). Months indicated are time scale relative to the flooding event.

Annotation of shotgun metagenomic reads from bulk water samples against the Comprehensive Antibiotic Resistance Database^52^ indicated that total ARGs per mL bulk water decreased from pre- to post-flood and then increased to near pre-flood abundances by ten months post-flood at both sites 1 and 5 (Fig. 2A). This decrease and subsequent increase suggests that the flood acted to “dilute” ARGs at both the pristine and impacted sites. Ten months of recovery, however, allowed sufficient time for ARG abundances to return to approximately pre-flood abundances.

A total of 277 ARG subtypes were identified across all samples, ranging from 77 to 155 subtypes per site (Fig. 2B,C). On average, trimethoprim resistance was the most common resistance type (39%), followed by multidrug (30%), polymyxin (11%), aminocoumarin (4%), peptide (4%), and tetracycline resistance (3%). The most common mechanism of resistance was efflux (46%), followed by antibiotic target replacement (39%), cell wall charge alteration (8%), antibiotic inactivation (5%), and molecular bypass (2%; Fig. S1).

**Figure 3:**
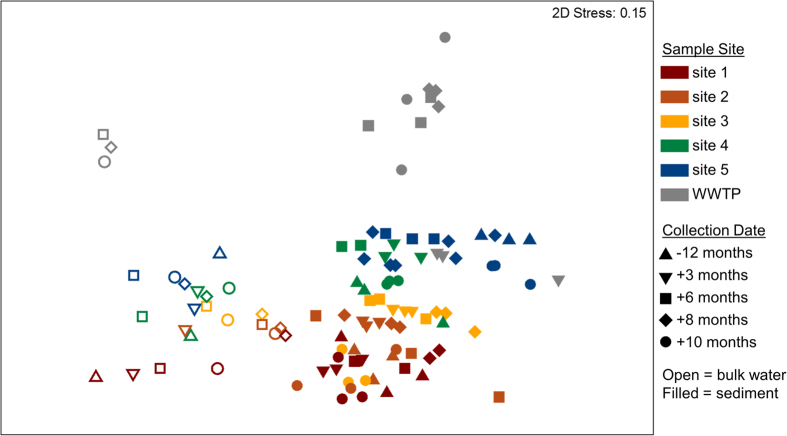
Beta Diversity plots of microbial community phylogenetic composition based on 16S rRNA gene amplicon sequencing of Poudre River bulk water (n = 1) and bed sediment (n = 3) samples coded by sample site and collection date based on distance matrixes generated using a jackknifed unweighted UniFrac metric.

The profile of individual ARG subtypes varied at both sites, indicating shifts in response to flooding and recovery, as illustrated by nonmetric multidimensional scaling (NMDS) analysis generated from a Bray-Curtis (BC) similarity matrix (Fig. 2D). Remarkably, NMDS analysis of ARGs indicated bulk water at both sites 1 and 5 shifted three months post-flood (site 1 BC = 58.3, site 5 BC = 58.9) but continued to shift to a unique profile by ten months post-flood (site 1 BC = 60.9, site 5 BC = 61.7). Interestingly, the shift observed at site 5 three months post-flood indicated similarity with site 1 pre-flood, though not statistically significantly (BC = 65.56), suggesting that the flood acted to “dilute” ARGs from the impacted site such that it resembled the pristine site, as is consistent with the decrease in total abundance of ARGs in post-flood samples (Fig. 2A). Surprisingly, while ARGs returned to pre-flood abundances, the ARG profile did not return to a pre-flood state, suggesting that the flooding may have disseminated new sources of ARGs that persisted at each site. While seasonal variation may also have contributed to the observed fluctuations, the overall stability of the bacterial community at each site across sample dates, relative to notable community variation across sites (Fig. 3), suggests that seasonal impact on biological variation was minimal.

### Potential for selection pressure indicated by co-occurrence of ARGs and antibiotics

**Figure 4:**
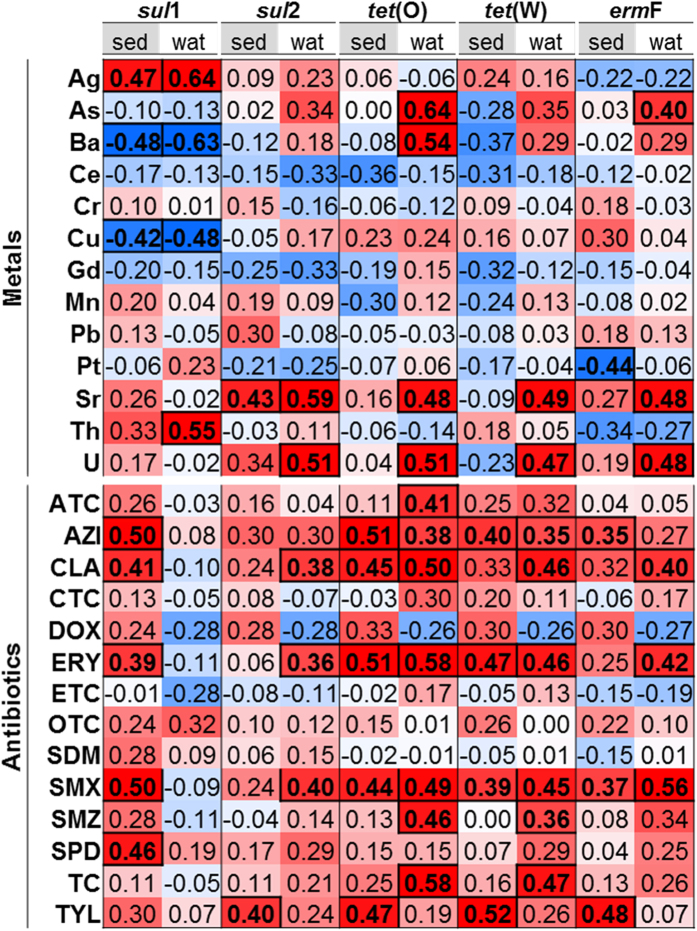
Spearman’s Rank Correlation Coefficient between abundance of ARGs in bed sediment (sed) and bulk water (wat) normalized to 16S rRNA genes, as determined by qPCR, and antibiotics or heavy metals. Statistically significant (p < 0.05) correlations indicated in bold. Blue shading indicates negative correlation and red shading indicates positive correlation. Antibiotics detected and included in the analysis were: anhydrotetracycline (ATC), azithromycin (AZI), clarithromycin (CLA), chlorotetracycline (CTC), doxycycline (DOX), erythromycin (ERY), 4-epitetracycline (ETC), oxytetracycline (OTC), sulfadimethoxine (SDM), sulfamethoxazole (SMX), sulfamethazine (SMZ), sulfadiazine (SPD), tetracycline (TC), and tylosin (TYL).

The potential role of antibiotics as selective agents influencing the re-establishment of ARGs during post-flood recovery was investigated by examining correlations between sulfonamide (*sul*1, *sul*2), tetracycline (*tet*(O), *tet*(W)), and macrolide (*erm*F) ARGs in bed sediment and bulk water, quantified using qPCR (Fig. S3), and 23 antibiotics (Table S2) in bulk water at all sites (Fig. 4). Due to the tendency of some antibiotics to lose antibacterial activity if they become sorbed to sediments or form complexes with substances such as humic acids^58^,^59^,^60^, analysis of antibiotics was limited to the bulk water. Correlations between antibiotics and ARGs in bulk water are hypothesized to be indicative of potential selective pressure while correlations between antibiotics in the bulk water and ARGs in sediment are likely to be indicative of deposition of bacteria that may have been subject to selection in the bulk water.

All ARGs demonstrated significant Spearman’s rank correlations with at least one antibiotic against which they conferred resistance, suggesting direct selection may be a key pressure shaping the resistome (Fig. 4). Bed sediment s*ul*1 exhibited moderate correlations with sulfamethoxazole and sulfadiazine (Spearman ρ = 0.4972, 0.4575; *p* = 0.0028, 0.0065), while bulk water *sul*2 was moderately correlated with sulfamethoxazole (ρ = 0.401.; *p* = 0.023). Bulk water *tet*(O) exhibited a moderate correlation with anhydrotetracycline and a strong correlation with tetracycline (ρ = 0.4133, 0.5764; *p* = 0.0187, 0.0006), while bulk water *tet*(W) was moderately correlated with tetracycline (ρ = 0.4657; *p* = 0.0072). *erm*F in bed sediment correlated weakly with azithromycin and moderately with tylosin (ρ = 0.3528, 0.4764; *p* = 0.0407, 0.0044) and in bulk water weakly with clarithromycin and moderately with erythromycin (ρ = 0.3999, 0.4172; *p* = 0.0234, 0.0175).

All ARGs identified were also found to significantly correlate with certain antibiotics against which they do not confer resistance (Fig. 4; p-values in Table S3), indicating potential for co-selection, which results from co-location of ARGs on the same genetic element, such as a plasmid, transposon, or integron; cross-resistance, which occurs when a single cellular response is capable of combatting multiple chemicals, such is the case with multidrug resistance pumps; or co-regulation, which occurs when two resistance regulation systems are transcriptionally linked^34^. Notably, numerous correlations were observed between antibiotics and *sul*1 and *tet*(O) ARGs. Bed sediment s*ul*1 exhibited a strong correlation with azithromycin, moderate correlation with clarithromycin, and weak correlation with erythromycin. Bulk water *tet*(O) exhibited a strong correlation with clarithromycin and erythromycin, moderate correlation with sulfamethoxazole, and sulfamethazine, and a weak correlation with azithromycin, while bed sediment *tet*(O) exhibited a strong correlation with azithromycin and erythromycin, and a moderate correlation with clarithromycin, sulfamethoxazole, and tylosin.

It is challenging to determine whether observed correlations are truly indicative of selective pressure or simply co-transport of antibiotics and ARGs from the same source. Covariation among antibiotics may also obscure true causative relationships of selection between genes and antimicrobial agents (Table S4). Based on metagenomic data, positive correlations were observed between MLS, rifampin, and fosfomycin ARGs and the antibiotics sulfamethazine (ρ = 0.8452, 0.8452, 0.8262, *p* = 0.0341, 0.0341, 0.0427) and clarithromycin (ρ = 0.8452, 0.8452, 0.8262, *p* = 0.0341, 0.0341, 0.0427).

While the concentrations of antibiotics observed in the Poudre River samples appear to be below minimum inhibitory concentrations, previous work has indicated that sublethal concentrations may aid in the dissemination of ARGs, via selection and other mechanisms. Gullberg *et al*.^61^ found that bacteria carrying plasmids with beta-lactam resistance genes were selected at concentrations of antibiotics and heavy metals nearly 140 times below reported minimum inhibitory concentration. Other studies have indicated that sublethal antibiotics may promote the dissemination of ARGs by stimulating horizontal gene transfer^29^,^30^.

### Potential for co-selective pressures exerted by heavy metals

All three mechanisms of co-selection described above also pertain to heavy metals. A previous study demonstrated that input of tetracycline resistant bacteria to the Poudre River and selection by tetracycline antibiotics was insufficient to explain the level of resistant bacteria present in the river, and identified co-selection by heavy metals as a likely source of resistant bacteria^62^. Therefore, the possibility of co-selection by heavy metals was investigated by examining correlations between 14 heavy metals (Table S5) and ARGs quantified by qPCR. *sul*1 exhibited strong Spearman’s rank correlations with several heavy metals. In bulk water and bed sediment, *sul*1 was positively correlated with silver (Spearman ρ = 0.6435, 0.4671; *p* = 0.0004, 0.0140) and negatively with both barium (ρ = −0.6315, −0.4804; *p* = 0.0005, 0.0112) and copper (ρ = −0.4806, −0.4229; *p* = 0.0.130, 0.0280). Strontium was positively correlated with bulk water and sediment *sul*2 (ρ = 0.5890, 0.4347; *p* = 0.0015, 0.0234) and bulk water *tet*(O), *tet*(W), and *erm*F (ρ = 0.4756, 0.4870, 0.4812; *p* = 0.0141, 0.0116, 0.0128). Uranium also exhibited positive correlations with *sul*2, *tet*(O), *tet*(W), and *erm*F in bulk water (ρ = 0.5123, 0.5092, 0.4695, 0.4825; *p* = 0.0075, 0.0079, 0.0155, 0.0125). Such robust correlations indicate a potential for co-selection by heavy metals, namely, by silver for *sul*1 and by strontium and uranium for *sul*2, *tet*(O), *tet*(W), and *erm*F. The unique behavior of *sul*1 compared to the other genes may result from the tendency for *sul*1 to be located on mobile genetic elements, such as class 1 integrons^63^,^64^,^65^. This characteristic may enable *sul*1 to become associated with various other ARGs and MRGs, making *sul*1 a prime candidate for co-selection. Copper has been previously identified as a metal that is likely to select for certain ARGs^14^,^66^, therefore its strong negative correlation with s*ul*1 was unexpected and may be indicative that copper selects for ARGs through mechanisms highly specific to certain conditions. Such negative correlations are not unprecedented, however, as a significant negative correlation was also observed previously between copper and sulfonamide ARGs in livestock lagoon water^67^. Though no significant positive correlations existed between copper and the five ARGs quantified by qPCR, copper was correlated with total resistance genes derived from the metagenomic data set for peptide (ρ = 0.8, *p* = 0.2), tetracycline (ρ = 0.8, *p* = 0.2), and sulfonamide (ρ = 0.6, *p* = 0.4) classes, though trends were not significant.

### Metagenomic scaffold associations reveals probable ARGs susceptible to co-resistance

**Figure 5:**
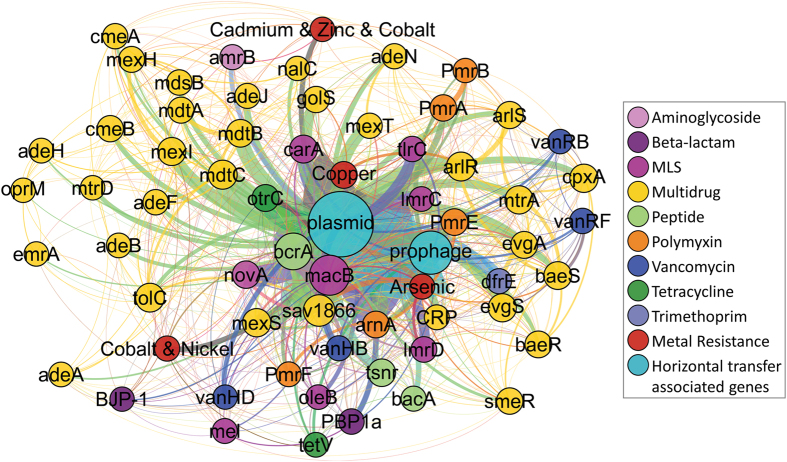
Co-occurrence of ARGs, MRGs, and genetic markers linked to mobile genetic elements on assembled scaffolds provides insight into which ARGs are candidates for co-selection or horizontal gene transfer. Network analysis indicating genetic proximity of ARGs, MRGs, plasmid sequences, and prophage sequences, based on co-occurrence of genes on scaffolds constructed using *de novo* assembly of shotgun metagenomic sequencing reads. Proximity of nodes and width of lines indicate frequency of associations between genes. Genetic markers with 20 or fewer instances of co-occurrence with other genes of interest were excluded from the network analysis rendering.

Network analysis was conducted to explore *de novo* assembled scaffolds for ARGs and MRGs physically co-located on DNA strands in order to identify genes that are likely candidates for co-resistance as a mechanism of co-selection (Fig. 5). Of a total of 52,556 scaffolds generated from all samples, 2,707 (5.2%) scaffolds contained more than one ARG and 347 (0.7%) scaffolds contained both ARGs and MRGs. Assembled scaffolds averaged 794 base pairs (bp) and reached a maximum length of 215,852 bp, ranging from 66,797 to 131,397 scaffolds per sample (Table S1). The most abundant ARG class associated with other ARGs revealed by the network analysis corresponded to efflux pumps (26.2%), multidrug resistance (12.3%), and macrolide/lincosamide/streptogramin (10.8%) resistance, while the most abundant co-located MRGs corresponded to copper and arsenic. The most frequent associations observed between genes were *mac*B and *bcr*A (0.16% of scaffolds), *otr*C and *bcr*A (0.06%), *Pmr*A and *Pmr*B (0.05%), and *sav*1866 and *bcr*A (0.04%) (Fig. 5). The ARGs that were subject to qPCR analysis, *sul*1, *sul*2, *tet*(O), *tet*(W), and *erm*F, were not found on any scaffolds with other ARGs or MRGs, which may stem from their relatively low abundance in the pool of metagenomic reads, which reduces likelihood of assembly (20% total reads were assembled).

### Role of horizontal gene transfer in shaping the resistome

Metagenomic data were searched for two families of mobile genetic elements, plasmids and prophages, as a proxy for potential for conjugation and transduction, respectively. Genes belonging to 32 different known plasmids were identified, along with genes corresponding to 65 different prophage genomes. A total of 3,912 (7.4%) scaffolds contained both ARGs and plasmid gene markers. Multiple ARGs were frequently found associated with plasmid markers on a single scaffold, with up to 11 ARGs found together on a single plasmid-associated scaffold. Four hundred and ninety-seven (0.9%) scaffolds contained one or more ARGs and prophage genetic markers. Network analysis revealed that the ARGs most frequently found on plasmid scaffolds were *mac*B (16.4% of plasmid scaffolds), *sav*1866 (5.2%), *mdt*C (3.1%), *otr*C (2.9%), *nov*A (2.5%), *arn*A (1.9%), and *mex*S (1.8%), while genes associated with copper (7.4%) and arsenic (2.3%) were the most common MRGs. *mac*B (20.5% of prophage scaffolds), *dfr*E (12.7%), and *arn*A (5.2%), were the ARGs most frequently found on prophage-associated scaffolds. The five ARGs examined by qPCR were not identified on any scaffolds associated with plasmids or prophages.

Although horizontal gene transfer is known to be an important mechanism in the spread of antibiotic resistance and provides an opportunity for pathogenic bacteria to acquire resistance from environmental bacteria^19^, it has been reported that it is a relatively rare event among soil bacteria and may be a relatively minor influence in shaping the resistome compared to phylogeny^20^,^68^. However, it has also been noted that plasmids carrying ARGs are significantly more likely to be conjugative than non-ARG carrying plasmids^69^ and broad host range plasmids were found to be capable of uptake by a highly diverse portion of the microbiome in a soil bacterial community study^70^. Although we could not precisely quantify the extent to which horizontal gene transfer shaped the resistome based on the present study, the numerous associations of plasmids and prophages with ARGs were striking, suggesting that it is a significant phenomenon in the riverine environment.

### Role of phylogeny in shaping the resistome

Based on jackknifed unweighted UniFrac distance, the microbial community composition observed in the bulk water of each site was distinct from that of the bed sediment (ANOSIM, R = 0.868, *p* = 0.001). Beta diversity plots, in which distance between samples is inversely proportional to similarity in phylogenetic composition, revealed that a clear shift in microbial community structure occurs along the anthropogenic gradient of the Poudre River. Sites clustered distinctly from each other, in bulk water (Fig. 3, ANOSIM, R = 0.488, *p* = 0.001) and more strongly in sediment (ANOSIM, R = 0.607, *p* = 0.001), but did not exhibit a discernible pattern when plotted based on sampling date for water (ANOSIM, R = 0.159, *p* = 0.033) or sediment (ANOSIM, R = 0.166, *p* = 0.001). The strong grouping by sample site indicates that anthropogenic influence on phylogeny is likely a more dominant controlling variable than seasonal variation, as well as for ARGs, as documented in previous studies of the Poudre River^7^, or even variation observed as a result of the flooding. This strong trend of microbial community variation along the anthropogenic gradient of the Poudre River suggests that adjacent land use is a key driver of sediment and bulk water microbial community. Another study also highlighted that watershed land use also plays a role in shaping the sediment microbial community of the Tongue River in Montana, USA^71^. The resilience of the microbial community in quickly rebounding to pre-flood conditions is consistent with another study that observed that following a whole-ecosystem mixing disturbance of a freshwater lake, the microbial community returned to pre-mixing composition and diversity in only 11 days^72^. Site 1 community composition was highly distinct from site 5 (ANOSIM, R = 0.929, *p* = 0.001) and WWTP effluent was dissimilar to all river sites (ANOSIM, R ≥ 0.947, *p* = 0.001). Site based similarity was less pronounced using weighted UniFrac distance (ANOSIM, R = 0.39, *p* = 0.001), which takes into account not only number of unique operational taxonomic units (OTUs) present, as with unweighted UniFrac, but abundance of each OTU. This weaker correlation indicates that rare species were particularly important in defining observed distinctions in microbial community among sites. *Proteobacteria, Bacteroidetes*, and *Cyanobacteria* were the most abundant phyla in the bulk water, with *Actinobacteria*, and *Verrucomicrobia* also contributing to more than 1% of phyla, on average (Fig. S4). Similarly, *Proteobacteria, Bacteroidetes, Cyanobacteria*, and *Verrucomicrobia* were the most dominant phyla in the sediment, with *Acidobacteria, Actinobacteria, Plantomycetes, Chloroflexi, Firmicutes, Nitrospirae*, and *Gemmatimonadetes* all contributing to greater than 1% of phyla (Fig. S5). Interestingly, the overall bulk water phylogeny was not correlated with ARG profiles (2STAGE, weighted UniFrac: Spearman’s ρ = −0.1) indicating that phylogeny alone may not be the most important factor controlling the profile of ARGs. This finding conflicts with previous studies that highlight host phylogeny as a key factor influencing antibiotic resistance in soil, sewage sludge, or agricultural environments^20^,^73^,^74^.

## Conclusions

This study uniquely characterized the impact of an extreme rainfall and flooding event on a riverine resistome using next-generation DNA sequencing. Following the flood, total bulk water ARGs decreased following the flood but recovered to near pre-flood abundances by ten months post-flood at both the pristine and impacted sites. Bulk water phylogeny did not correlate with ARG profiles, but sediment phylogeny varied according to the river’s anthropogenic gradient. Quantitative monitoring of ARGs and two classes of selective agents, antibiotics and heavy metals, was suggestive of selective pressure in the reestablishment of the resistome following the flood. Additionally, we identified ARGs found on assembled metagenomic scaffolds associated with other ARGs, MRGs, and mobile genetic element genes as likely candidates for co-selection or horizontal gene transfer. The results of this study help elucidate the mechanisms contributing to proliferation of ARGs in surface water and inform management strategies limiting anthropogenic contributions of ARGs to the environment.

## Additional Information

**How to cite this article**: Garner, E. *et al*. Metagenomic profiling of historic Colorado Front Range flood impact on distribution of riverine antibiotic resistance genes. *Sci. Rep.* **6**, 38432; doi: 10.1038/srep38432 (2016).

**Publisher's note:** Springer Nature remains neutral with regard to jurisdictional claims in published maps and institutional affiliations.

## Supplementary Material

Supplementary Information

Supplementary Table S2

Supplementary Table S3

Supplementary Table S4

Supplementary Table S5
