# Supplementary material for: Metagenomic profiling of historic Colorado Front Range flood impact on distribution of riverine antibiotic resistance genes
Source: Sci Rep. 2016 Dec 5;6:38432. doi: 10.1038/srep38432 (PMC5137141; doi:10.1038/srep38432)
Supplement: Supplementary Information [file srep38432-s1.pdf]

Supplementary Information

Title: Metagenomic profiling of historic Colorado Front Range flood impact on distribution of riverine antibiotic resistance genes

Authors:

Emily Garner[1], Joshua S. Wallace[2], Gustavo Arango Argoty[3], Caitlin Wilkinson[1], Nicole Fahrenfeld[4], Lenwood S. Heath[3], Liqing Zhang[3], Mazdak Arabi[5], Diana S. Aga[2], and Amy Pruden[1]*

[1]Department of Civil and Environmental Engineering, Virginia Tech, Blacksburg, VA 24061

[2]Department of Chemistry, University at Buffalo, The State University of New York, Buffalo, NY 14260

[3]Department of Computer Science, Virginia Tech, Blacksburg, VA 24061

[4]Department of Civil and Environmental Engineering, Rutgers University, Piscataway, NJ 08854

[5]Department of Civil and Environmental Engineering, Colorado State University, Fort Collins, CO, 80523

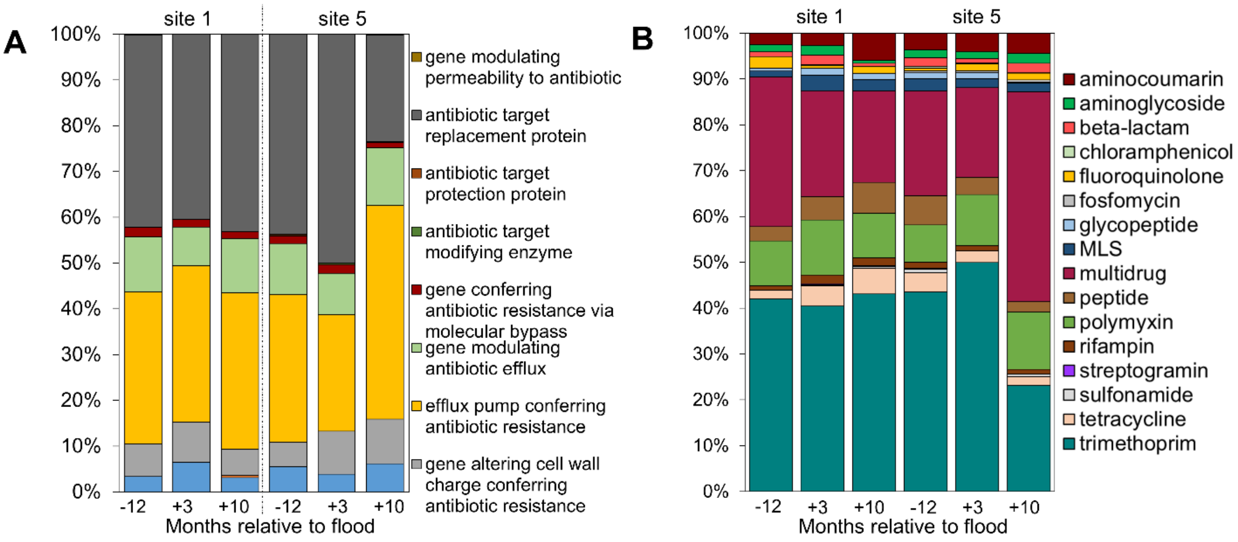

Figure S1: Relative abundance of ARGs as determined by metagenomic analysis of site 1 and site 5 Poudre River bulk water samples. (A) Mechanisms of antibiotic resistance and (B) classes of ARGs determined by metagenomic analysis and annotation against the Comprehensive Antibiotic Resistance Database.

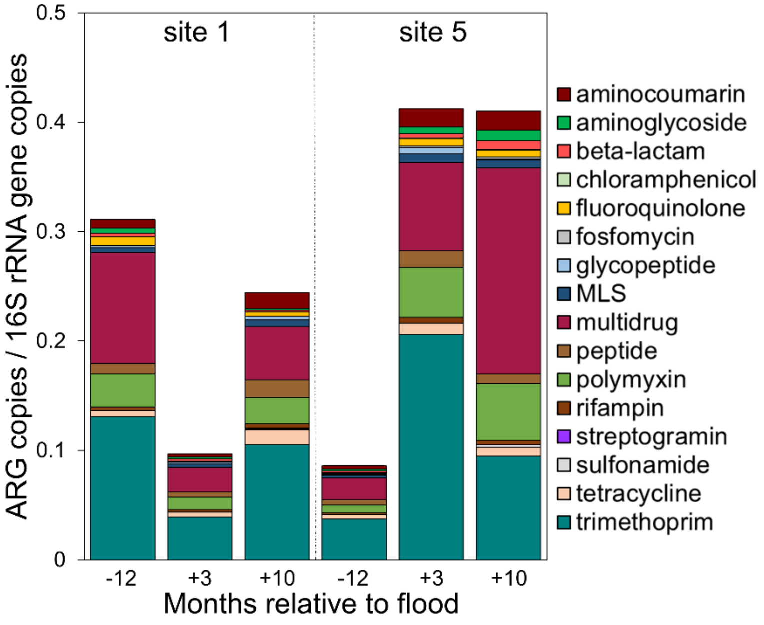

Figure S2: Relative abundance (ARG copies / 16S rRNA gene copies) of ARGs as determined by metagenomic analysis of site 1 and site 5 Poudre River bulk water samples

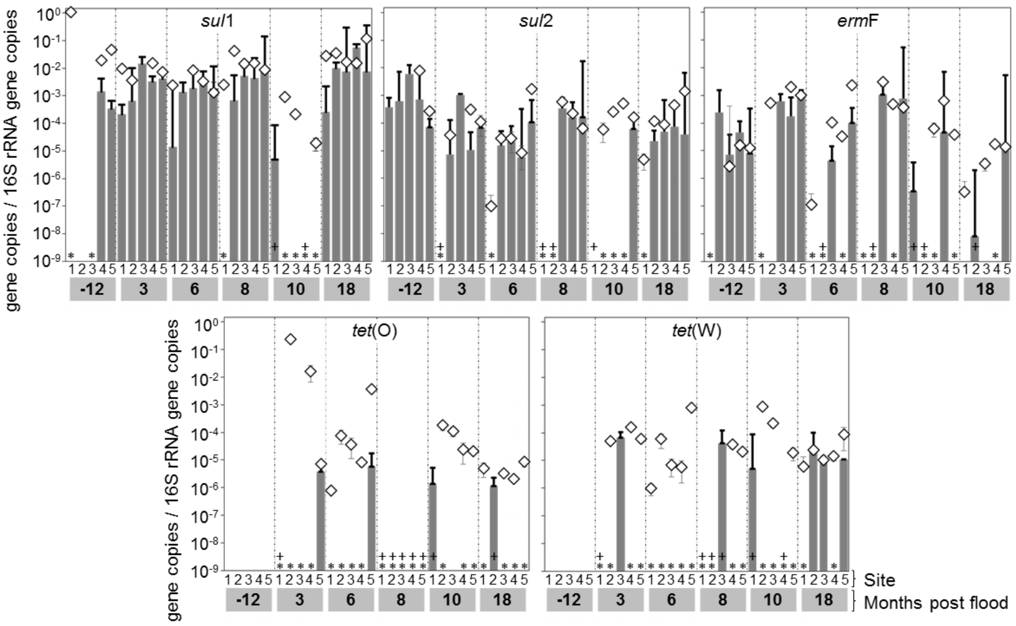

Figure S3: Quantification of select ARGs by quantitative polymerase chain reaction (qPCR) in Poudre River sediment (bars) and bulk water (points), normalized to 16S rRNA genes at 12 months (-12) before the flooding occurred and at five time points following the flooding. X-axis indicates sites and months relative to the flooding event. (*) indicates gene detected below quantification limit in sediment and (+) in water. Error bars represent standard deviation of triplicate qPCR measurements in water and standard deviation of triplicate samples in sediment.

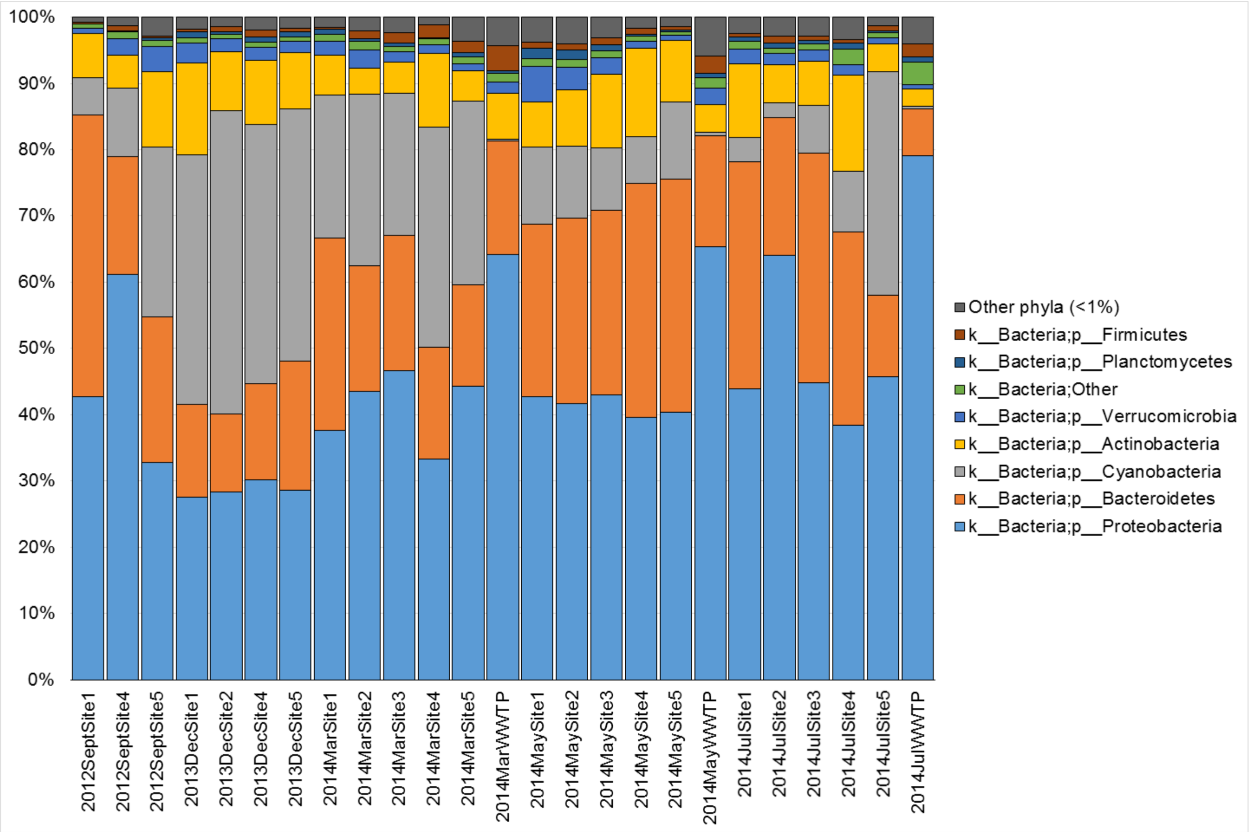

Figure S4: Phyla accounting for greater than 1% of the total OTUs in bulk water, determined by 16S rRNA gene sequencing.

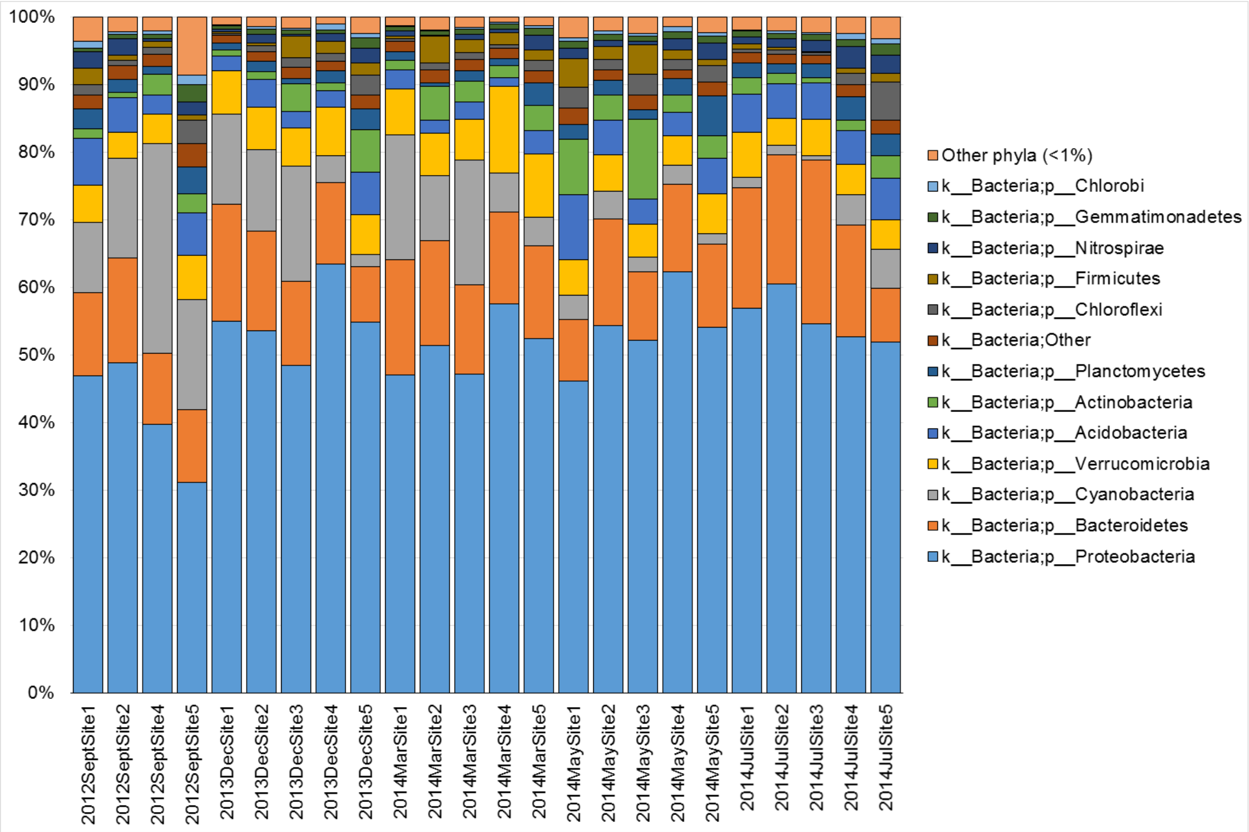

Figure S5: Phyla accounting for greater than 1% of the total OTUs in bulk water, determined by 16S rRNA gene sequencing. Triplicate sediment samples were sequenced separately and results averaged.

	*sul*1		*sul*2		*tet*(O)		*tet*(W)		*erm*F	
	sed	wat	sed	wat	sed	wat	sed	wat	sed	wat
Chlorophyll a	0.3088	0.1764	**0.4649**	0.363	0.0034	0.0304	-0.0655	0.0983	0.2332	0.3054
Dissolved Oxygen	**-0.4757**	**-0.4842**	-0.2325	-0.2285	-0.2078	0.1339	**-0.472**	-0.1751	-0.0835	0.0616
pH	-0.2775	-0.27	-0.1149	0.012	**-0.4998**	0.1495	**-0.6052**	-0.2384	-0.1592	0.2058
Salinity	**0.4087**	0.1502	**0.5256**	**0.4834**	0.2084	0.2838	0.0775	0.3121	**0.4145**	**0.4227**
Specific Conductivity	0.2872	0.0516	**0.4329**	0.3117	0.0845	0.1342	-0.1389	0.0175	0.3243	**0.3949**
Temperature	-0.3435	-0.0858	0.2143	0.3267	-0.1941	-0.0232	-0.1006	-0.0251	0.3192	0.1112

Figure S6: Spearman's Rank Correlation Coefficient between abundance of ARGs normalized to 16S rRNA genes, as determined by qPCR, and water quality paramters. Statistically significant ($p<0.05$) correlations are indicated in bold.

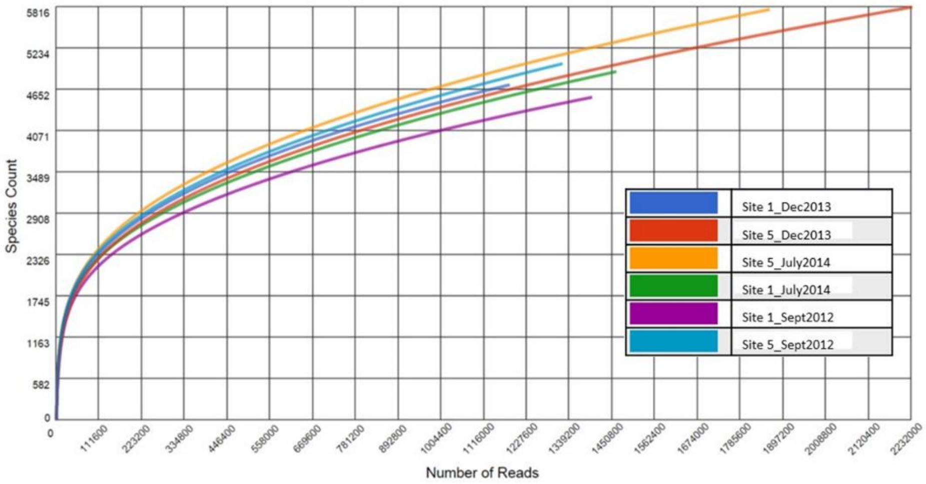

Figure S7: Rarefaction curves for metagenomic samples.

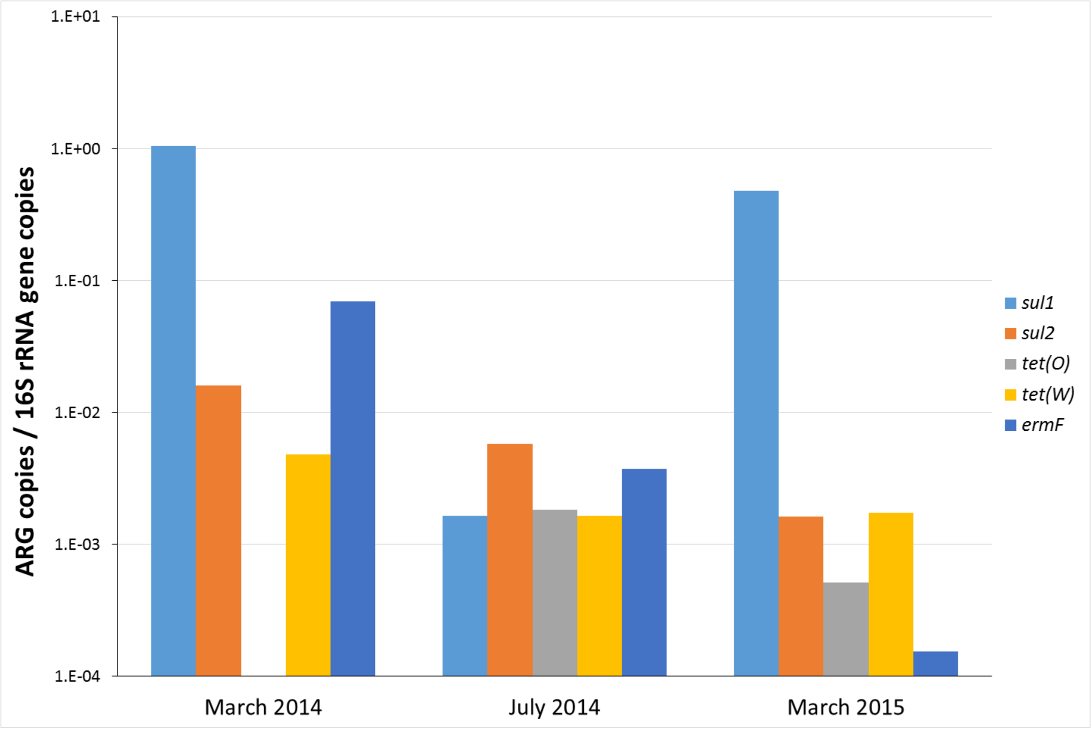

Figure S8: ARG copies determined by qPCR in WWTP effluent, normalized to 16S rRNA gene copies.

Table S1: Characteristics of metagenomic data. All sequences have been deposited in MG-RAST under project name "Fate and Transport of Antibiotics and Antibiotic Resistance Genes during Historic Colorado Flood."

Sample Name	*MG-RAST ID (unassembled)*	*# Reads (unassembled)*	*# scaffolds*	*Average scaffold length (bp)*	*Maximum scaffold length (bp)*
Poudre_WSite1_Sept2012	4628878.3	1,397,640	131,397	1005	137,041
Poudre_WSite1_Dec2013	4628876.3	1,182,555	81,537	644	35,373
Poudre_WSite1_July2014	4628877.3	1,460,502	66,797	794	78,321
Poudre_WSite5_Sept2012	4628881.3	1,320,893	86,492	625	153,801
Poudre_WSite5_Dec2013	4628879.3	2,232,495	96,292	751	91,128
Poudre_WSite5_July2014	4628880.3	1,860,238	97,413	829	215,852

Table S2: Antibiotic concentrations in Poudre River bulk water (ng/L). Standard deviation of replicate samples denoted in parentheses and months indicate months post-flood. "W" denotes Wastewater Treatment Plant samples. Antibiotics abbreviations are denoted as follows: anhydrotetracycline (ATC), azithromycin (AZI), clarithromycin (CLA), chlorotetracycline (CTC), doxycycline (DOX), erythromycin (ERY), 4-epitetracycline (ETC), oxytetracycline (OTC), sulfamerazine (SMR), sulfamethoxazole (SMX), sulfamethazine (SMZ), sulfadiazine (SPD), tetracycline (TC), and tylosin (TYL). Sulfameter, sulfamethiazole, sulfamerazine, sulfachloropyridazine, sulfathiazole, roxithromycin, spiramycin, 4-epichlorotetracycline, anhydrochlorotetracycline, demeclocycline (surrogate), minocycline (internal standard), phenyl-13C6-sulfamethazine (13C6-SMZ), d4-sulfamethoxazole (d4-SMX), N-methyl 13C-erythromycin, and d10-carbamazepine (internal standard) were not detected in any samples.

Table S3: p-values for Kruskal-Wallis rank sum tests for correlations between ARGs and antibiotics of metals. Significant ($p<0.05$) values indicated in bold.

Table S4: Correlation matrix using Spearman's rank sum correlation coefficients. Statistically significant ($p<0.05$) correlations are indicated in bold.

Table S5: Metal concentrations in Poudre River bulk water (µg/L). Standard deviation of replicate samples denoted in parentheses.
